# Increased functional connectivity of white-matter in myotonic dystrophy type 1

**DOI:** 10.3389/fnins.2022.953742

**Published:** 2022-08-01

**Authors:** Jing Li, Jie Li, Pei Huang, Li-Na Huang, Qing-Guo Ding, Linlin Zhan, Mengting Li, Jiaxi Zhang, Hongqiang Zhang, Lulu Cheng, Huayun Li, Dong-Qiang Liu, Hai-Yan Zhou, Xi-Ze Jia

**Affiliations:** ^1^School of Teacher Education, Zhejiang Normal University, Jinhua, China; ^2^Key Laboratory of Intelligent Education Technology and Application of Zhejiang Province, Zhejiang Normal University, Jinhua, China; ^3^Research Center of Brain and Cognitive Neuroscience, Liaoning Normal University, Dalian, China; ^4^Key Laboratory of Brain and Cognitive Neuroscience, Dalian, China; ^5^Department of Neurology & Institute of Neurology, Ruijin Hospital, Shanghai Jiao Tong University School of Medicine, Shanghai, China; ^6^Department of Radiology, Changshu No. 2 People’s Hospital, The Affiliated Changshu Hospital of Xuzhou Medical University, Changshu, China; ^7^Faculty of Western Languages, Heilongjiang University, Harbin, China; ^8^School of Foreign Studies, China University of Petroleum, Qingdao, China; ^9^Shanghai Center for Research in English Language Education, Shanghai International Studies University, Shanghai, China

**Keywords:** myotonic dystrophy type 1, resting-state fMRI, white-matter functional networks, white-matter, functional connectivity

## Abstract

**Background:**

Myotonic dystrophy type 1 (DM1) is the most common and dominant inherited neuromuscular dystrophy disease in adults, involving multiple organs, including the brain. Although structural measurements showed that DM1 is predominantly associated with white-matter damage, they failed to reveal the dysfunction of the white-matter. Recent studies have demonstrated that the functional activity of white-matter is of great significance and has given us insights into revealing the mechanisms of brain disorders.

**Materials and methods:**

Using resting-state fMRI data, we adopted a clustering analysis to identify the white-matter functional networks and calculated functional connectivity between these networks in 16 DM1 patients and 18 healthy controls (HCs). A two-sample *t*-test was conducted between the two groups. Partial correlation analyzes were performed between the altered white-matter FC and clinical MMSE or HAMD scores.

**Results:**

We identified 13 white-matter functional networks by clustering analysis. These white-matter functional networks can be divided into a three-layer network (superficial, middle, and deep) according to their spatial distribution. Compared to HCs, DM1 patients showed increased FC within intra-layer white-matter and inter-layer white-matter networks. For intra-layer networks, the increased FC was mainly located in the inferior longitudinal fasciculus, prefrontal cortex, and corpus callosum networks. For inter-layer networks, the increased FC of DM1 patients is mainly located in the superior corona radiata and deep networks.

**Conclusion:**

Results demonstrated the abnormalities of white-matter functional connectivity in DM1 located in both intra-layer and inter-layer white-matter networks and suggested that the pathophysiology mechanism of DM1 may be related to the white-matter functional dysconnectivity. Furthermore, it may facilitate the treatment development of DM1.

## Introduction

Myotonic dystrophy type 1 (DM1) is the most common and dominant inherited neuromuscular dystrophy disease in adults (Emery, 1991). This multi-systemic disorder is characterized by muscular impairment but affects different organs, including the brain (Meola and Sansone, 2007). Neuroimaging studies revealed widespread abnormalities in the brain structure of DM1 patients, particularly white-matter damage (Minnerop et al., 2011; Caso et al., 2014; Wozniak et al., 2014; Baldanzi et al., 2016; Gourdon and Meola, 2017). The abnormalities of white-matter in DM1 patients manifested have increased white-matter hyperintensity load, decreased microstructural integrity, and significant diffusivity alterations (Cabada et al., 2017; Lopez-Titla et al., 2021). These white-matter abnormalities in DM1 patients may be associated with episodic memory, executive function, and visuo-spatial impairments, which impact the quality of their life (Meola et al., 1996; Modoni et al., 2008; Rakocevic-Stojanovic et al., 2014; Gallais et al., 2015; Schneider-Gold et al., 2015; Baldanzi et al., 2016; Okkersen et al., 2017). These findings provided evidence for subtle white-matter changes in DM1 patients (Minnerop et al., 2011).

However, these studies only explored the details of white-matter architectures using the diffusion tensor imaging (DTI) technique, they cannot accurately reflect changes in brain function. Resting-state functional magnetic resonance imaging (rs-fMRI) techniques, based on blood oxygen level-dependent (BOLD) signals, are widely used for investigating brain functional activity in brain diseases (Biswal et al., 1995; Meda et al., 2014). In the past, most fMRI studies focused on BOLD signal changes in the gray-matter while ignoring the white-matter since it has limited postsynaptic potentials that cause BOLD signals (Logothetis et al., 2001). Thus, they considered white-matter a nuisance regressor and removed the BOLD signals of white-matter despite taking up half of the brain volume (Behzadi et al., 2007; Caballero-Gaudes and Reynolds, 2017).

Recent studies, however, showed strong evidence that functional information in white-matter could be feasibly and reliably revealed by BOLD-fMRI, ranging from multistate (tasks and resting-state) to multimodal MRI (fMRI and DTI) findings for different brain types (healthy, patients, and primate monkeys). The fMRI activation in white-matter (internal capsule and corpus callosum) can be identified across perceptual, language, and motor tasks (Fabri et al., 2011; Gawryluk et al., 2011, 2014; Fabri and Polonara, 2013). Within these white-matter tracts, studies further confirmed that the changes in low-frequency BOLD fluctuations could be modulated by various stimuli and indicated that they are involved in neural coding and information processing (Marussich et al., 2017; Wu et al., 2017; Ding et al., 2018; Huang et al., 2018; Li et al., 2019b). In addition to task-based fMRI, rs-fMRI studies also found that BOLD signals within white-matter were not random noise but carried functional information and showed an intrinsic functional organization (Ding et al., 2013, 2016, 2018; Peer et al., 2017; Li et al., 2019a). Moreover, using clustering methods, BOLD-fMRI signals in white-matter can be organized into large-scale functional networks, which showed a similar pattern to the white-matter tracts obtained from DTI in healthy participants (Marussich et al., 2017; Peer et al., 2017). Furthermore, in primate monkeys, Wu and his colleagues reconfirmed the significance of functional activities in white-matter (Wu et al., 2019). Based on these evidence, the white-matter functional networks thus were widely used to explore the neural mechanisms of neuropsychiatric disorders (Jiang et al., 2019b; Bu et al., 2020; Fan et al., 2020; Li et al., 2020; Lu et al., 2021). For instance, Jiang et al. found that patients with schizophrenia showed increased FC in the perception-motor white-matter network (Jiang et al., 2019a). More recently, the abnormalities of FC in the white-matter functional network were also revealed in other diseases, including epilepsy, attention deficit hyperactivity disorder, bipolar disorder, schizophrenia, Parkinson’s, and depression (Jiang et al., 2019a,b; Bu et al., 2020; Fan et al., 2020; Li et al., 2020; Lu et al., 2021) and even in participants with orthodontic pain or jet lag (Zhang et al., 2021a,b). Taken together, these studies provided strong evidence of the existence of functional brain activity in the white-matter and suggested that the functional information from white-matter can be detected by fMRI. We thus can construct white-matter functional networks and estimate functional connectivity in white-matter in a suitable way (Ding et al., 2013, 2016; Ji et al., 2017; Marussich et al., 2017; Huang et al., 2018; Li et al., 2019a).

As a predominant white-matter disease, only a few studies suggested that DM1 patients had increased FC in the default mode network (DMN) and theory-of-mind (ToM) network related to the higher-level cognitive function (Serra et al., 2014, 2016). The aberrant integration properties of brain functional networks, primarily focused on the gray-matter, provided evidence for the complex pathophysiological mechanisms of DM1 (Serra et al., 2014, 2016). However, they overlooked the significance of the white-matter functional activity. We remain unclear about the contribution of white-matter function in the pathophysiology of DM1. Therefore, we anticipated that exploring the altered white-matter functional networks in DM1 patients may contribute to understanding its underlying pathological mechanisms.

This study aimed to construct white-matter functional networks by a clustering method and then evaluate FC between these networks in 16 DM1 patients and 18 HCs. We expected to reveal the role of white-matter functional networks in DM1 patients by comparing the differences in FC between the two groups. To our knowledge, this study is a first attempt to examine the white-matter functional networks abnormalities in DM1 to better understand the pathological mechanism of myotonic dystrophy.

## Materials and methods

### Participants

Diagnosis of DM1 was based on clinical features, together with electromyographic evidence of myopathy and myotonia. Eighteen DM1 patients were enrolled and genetically confirmed. After two patients were excluded for large head motion, two groups were matched on age, gender, and education. This study included 16 patients with DM1 (gender: 10 males and 6 females; age: 48.00 ± 14.14 years) and eighteen healthy controls (gender: 9 males and 9 females; age: 41.50 ± 10.65 years). The inclusion criteria for patients were as follows: (1) right-handed according to Edinburgh Handedness Inventory (EHI) (Oldfield, 1971); (2) age ranging from 20 to 80 years; (3) Confirmed by genetic testing. Genomic DNA was isolated from peripheral blood, with consent from each individual, using the Wizard genomic DNA purification kit (Promega, Madison, WI, United States). Polymerase chain reaction (PCR) with primers DM1-F and DM1-R was used to amplify the region of the DMPK gene, including the CTG repeat. The GeneScan analysis program on an automated sequencer (ABI Prism 3130 Genetic Analyzer, Applied Biosystems) was used to estimate the allele size. Southern blot analysis was performed to detect the larger allele of the CTG expansion. CTG expansion equal to or larger than 50 was considered positive. Exclusion criteria included: (1) history of drug and alcohol abuse; (2) brain damage, such as head trauma or history of stroke; (3) other diseases that cause muscle weakness, such as myositis, myasthenia gravis, peripheral neuropathy, etc.; (4) MRI incompatibility. All the participants were evaluated by neurologists. Cognition and depression were assessed by Mini-mental state examination (MMSE) and Hamilton depression scale-17 (HAMD-17).

All participants were fully informed and signed written consent forms. This study was approved by the ethics committee of Ruijin Hospital Affiliated to Shanghai Jiao Tong University School of Medicine, and registered on the Chinese clinical trial registry (ChiCTR2000032978).

### Image acquisition

All participants were instructed to remain awake, close their eyes, and think of nothing while underwent Tesla GE Medical System (GE Healthcare, Little Chalfont, United Kingdom) scans. High-resolution T1-weighted anatomical images were acquired by a three-dimensional fast spoiled gradient-echo (T1-3D FSPGR) sequence. The main scanning parameters include: repetition time (TR) = 5.5 ms, echo time (TE) = 1.7 ms, flip angle (FA) = 12°, matrix size = 256 × 256, and slice thickness = 1 mm. Resting-state functional images were obtained using an echo-planar imaging (EPI) sequence. The main scanning parameters were: TR = 2,000 ms, TE = 30 ms, FA = 90°, matrix size = 64 × 64, slice thickness = 4 mm, slice number = 35, and scanning time = 420 s (210 volumes). We confirmed that all participants did not fall asleep during the scanning.

### Resting-state fMRI data preprocessing

The preprocessing of resting-state data was performed using the Statistical Parametric Mapping software (SPM12, www.fil.ion.ucl.ac.uk/spm), Data Processing & Analysis for Brain Imaging (DPABI_V4.1^1^), and open MATLAB scripts^2^, and the data preprocessing steps were similar to prior studies (Peer et al., 2017; Jiang et al., 2019a). Briefly, the structural image, which was co-registered to functional images after motion correction, was segmented into WM, GM, and cerebrospinal fluid (CSF) using the SPM12’s New Segment algorithm and then spatially normalized to the Montreal Neurological Institute standard (MNI) template with the DARTEL algorithm (Ashburner, 2007).

The functional image preprocessing steps consisted of the following steps: (1) Removal of the first 10 time points. (2) Slice-time correction. (3) Realignment. According to previous studies, two participants with maximum head motion > 2 mm or 2° were excluded (Jiang et al., 2019a,b; Zhang et al., 2021a). (4) Nuisance regression (regressors: linear trends, 24-parameter motion correction (Friston et al., 1996) and the mean CSF signals). The WM matter and global brain signals were not regressed out for avoiding eliminating signals of interest. (5) Temporal scrubbing was also performed with motion “spikes” (framewise displacement (FD) > 1) as separate repressors. (6) Band-pass filtering (0.01–0.15 Hz). (7) Spatial smoothing (full width at half maximum (FWHM) = 4 mm). Notably, smoothing was performed separately within white-matter functional images to avoid mixing signals of white-matter and gray-matter. Specifically, for each participant, T1 segmentation images were co-registered to the functional space to identify white-matter or gray-matter masks (To show the segmentation effects of patients, we provided the segmentation effect maps of seven DM1 patients in the Supplementary material; see Supplementary Figures S1–S7), and then the individual functional images were smoothed respectively on the two masks. In this study, we used only the smoothed data on the WM mask. (8) Normalization. The images were normalized to a standard EPI template with a voxel size of 3 × 3 × 3 mm using the DARTEL algorithm.

### Clustering white-matter networks in the white-matter mask

The construction of WM functional networks was similar to previous studies (Peer et al., 2017) and briefly described in the following steps. First, group-level unified white-matter and gray-matter masks were obtained from the T1 segmentation images. For each subject, we identified each voxel as WM, GM, or CSF based on its maximum probability from the segmentation results, and thus obtained individual white-matter, gray-matter, and CSF masks. These masks were averaged across all the participants, and the percentage of each voxel classified as WM or GM was calculated. Voxels with a percentage > 60% were then identified as the group-level WM mask (Peer et al., 2017). Then, the subcortical areas based on the Harvard-Oxford Atlas (Desikan et al., 2006) were removed from the WM mask. This mask was co-registered to the functional space and resampled for the processing of the functional image.

Second, for each subject, the voxel-level correlation matrix was constructed by calculating Pearson’s correlation coefficients between voxels restricted in the group-level unified WM mask. To reduce the computational complexity, we subsampled 12,747 voxels in the white-matter mask to 3,201 nodes with an interchanging grid strategy (Craddock et al., 2012). In detail, any second voxels along the rows and columns were taken and then moved by one between the two slices. Pearson’s correlation coefficients between each WM voxel and subsampled node were computed and resulted in a correlation pattern (12,747 × 3,201 matrix) for each subject.

**FIGURE 1:**
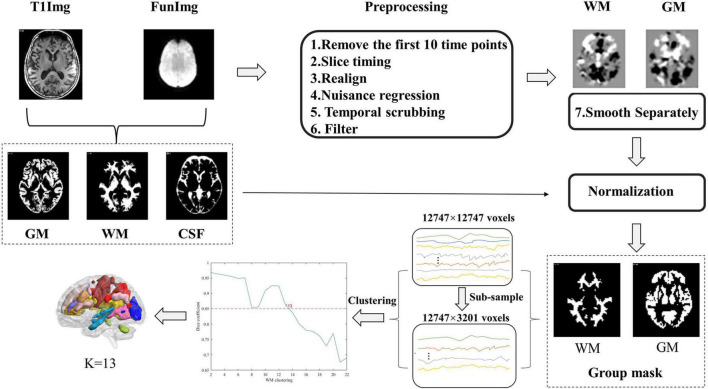
Flow chart of the processing of the resting-state functional magnetic resonance imaging (Rs-Fmri) data and the clustering of white-matter.

Finally, the clustering approach was used to identify the white-matter networks. K-means clustering (distance metric-correlation, 10 replicates) was performed on the averaged correlation matrices. The correlation matrix was first averaged across the participants in each group and then averaged again between the DM1 and HCs groups. To obtain the most stable number of networks, we measured the stability of the number of each cluster according to the method previously described (Buckner et al., 2011). The number of clusters ranges from 2 to 22. We randomly divided the whole connectivity matrix (12,747 × 3,201) into 4-folds (12,747 × 800). For each number of clusters, the same clustering computation was performed on each fold separately. To measure the similarity between the clustering in 4-folds, an adjacency matrix and averaged Dice?s coefficient across these folds were calculated for comparison, and the stability of the number of clusters was assessed. The steps of the construction of functional white-matter networks are shown in Figure 1.

### Functional connectivity of white-matter networks

To evaluate the functional connectivity between each identified WM network, we extracted the average time courses from each WM network by averaging across all voxels belonging to one network for each participant. The Pearson’s correlation was computed for each subject between any two WM networks and then transformed to the Fisher’s *z*-score. The two-sample *t*-test was performed on the *z*-score of Pearson’s correlation coefficient to identify the differences between DM1 and HCs groups. Age, education, gender and mean FD were regressed as covariates in the two-sample *t*-test to avoid their influence. Notably, the *p*-value was estimated by adopting a permutation test with the number set at 1,000. The statistical significance level was set at *p* < 0.05, and network-based statistic (NBS) was adjusted for multiple testing. A previous study showed that connected subnetworks of edges, showing a particular effect of a size larger than which would be expected by chance, can be identified by this NBS statistical method (Zalesky et al., 2010).

### Correlations between abnormal functional connections and clinical variables

We further investigated the relationships between clinical variables (disease duration, MMSE, and HAMD-17 scores) and altered functional connectivity. The partial correlation analyzes were performed for the DM1 group, controlling for age, gender, education, and mean FD.

## Result

### Demographic and clinical characteristics

The demographics and clinical data are shown in Table 1. There were no significant between-group differences in age (*p* = 0.11), sex distribution (*p* = 0.51), or educational level (*p* = 0.12). DM1 patients showed significantly lower scores in MMSE *(p* < 0.001) and higher scores in HAMD-17 (*p* < 0.001) than HCs. We also computed mean the Framewise Displacement (mean FD) (Power et al., 2012) and the mean frame-to-frame root mean square (RMS) motion of each subject, respectively (Van Dijk et al., 2012). The former is calculated by summing the absolute value of displacement changes in the *x*, *y*, and *z* directions and rotational changes about those three axes (Power et al., 2012), the latter calculated by the mean frame-to-frame root mean square (RMS) motion in *x*, *y*, and *z* directions (Van Dijk et al., 2012). Although mean FD was slightly significantly higher in the DM1 group relative to HCs (Power et al., 2012), there was no RMS relative motion difference between DM1 and HCs individuals (*p* = 0.48). We also accounted for motion by including mean FD as a group-level covariate.

**TABLE 1:** Participant demographics.

	DM1 (*N* = 16) (Mean ± SD)	HCs (*N* = 18) (Mean ± SD)	Group comparison (P-value)
Age (years)	48 ± 14.14	41 ± 10.65	0.11^a^
Education (years)	13 ± 3.16	14.55 ± 2.52	0.12^a^
Gender (male/female)	10/6	9/9	0.51^b^
Mean FD	0.11 ± 0.04	0.08 ± 0.03	0.03^a^
Mean relative RMS	0.02 ± 0.01	0.01 ± 0.01	0.43^a^
Mean RMS	0.21 ± 0.11	0.34 ± 0.24	0.06^a^
Duration	6.19 ± 4.82	–	–
HAMD-17	6.00 ± 4.23	2.06 ± 0.80	<0.001
MMSE	27.06 ± 1.84	29.17 ± 0.92	<0.001

DM1, Myotonic Dystrophy Type 1; HCs, healthy controls; Mean FD, framewise displacement computed following Power; RMS Mean Displacement, frame-to-frame root mean square motion in the *x*, *y*, and *z* directions measured following Van Dijk; MMSE, Mini-Mental State Examination; HAMD-17, Hamilton Depression Scale-17.

^a^Two-sample *t*-test.

^b^Chi-square test.

### White-matter functional networks

**FIGURE 2:**
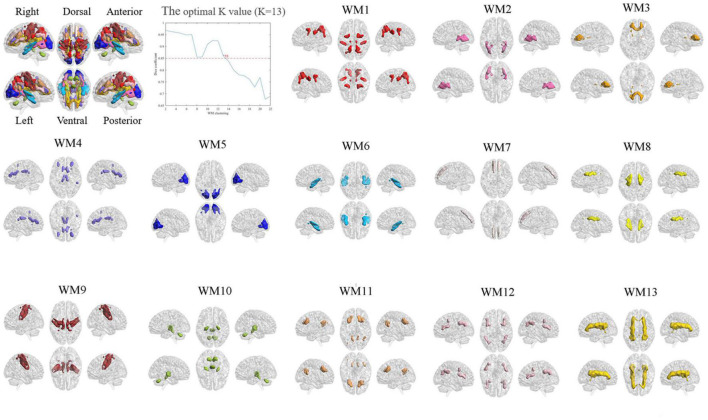
The optimal K-value and functional white-matter networks. WM1 (superior longitudinal fasciculus network), WM2 (inferior longitudinal fasciculus network), WM3 (anterior corpus callosum network), WM4 (corpus callosum network), WM5 (occipital network), WM6 (inferior temporal), WM7 (prefrontal network), WM8 (superior corona radiata network), WM9 (post-central network), WM10 (cerebellar network), WM11 (posterior callosum network), WM12 (inferior frontoparietal network), and WM13 (deep network).

**FIGURE 3:**
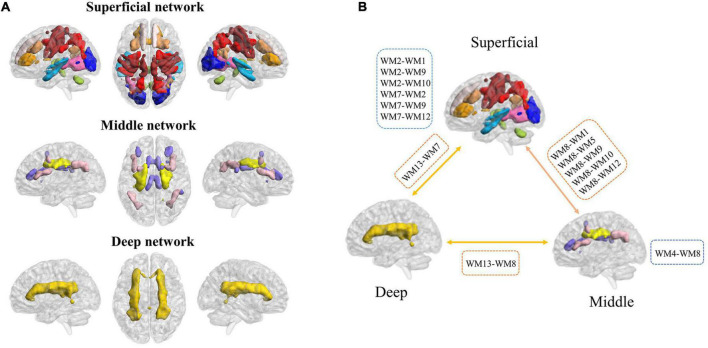
Three layers of functional white-matter networks. **(A)** Three layers were defined based on the spatial distance to the gray-matter (superficial, middle, and deep). **(B)** The increased intra-layer and inter-layer connections of functional white-matter networks showed in myotonic dystrophy type 1 (DM1). The blue dashed boxes represent increased FC between functional white-matter networks in the intra-layer, while the orange dashed boxes represent increased FC between functional white-matter networks in the inter-layer.

To evaluate the stability of the number of functional white-matter networks, we calculated the Dice’s coefficient across four folds. The result showed that *K* = 13 is the largest number with high stability (Dice’s coefficient > 0.85). Therefore, 13 white-matter networks were used in the subsequent analyzes. The spatial visualization and the detailed information of these networks are presented in Figure 2 and Table 2 separately. According to the spatial location, we named them WM1 (superior longitudinal fasciculus network), WM2 (inferior longitudinal fasciculus network), WM3 (anterior corpus callosum network), WM4 (corpus callosum network), WM5 (occipital network), WM6 (inferior temporal), WM7 (prefrontal network), WM8 (superior corona radiata network), WM9 (post-central network), WM10 (cerebellar network), WM11 (posterior callosum network), WM12 (inferior frontoparietal network), and WM13 (deep network). The present networks WM3, WM4, WM5, WM7, WM8, WM9, and WM13 are similar to the results of the previous researches (Jiang et al., 2019a; Li et al., 2022), while the other WM networks can also be obtained in some studies (Peer et al., 2017; Zhang et al., 2021b). Consistent with previous studies, these WM networks can be divided into three layers (superficial, middle, and deep) (Jiang et al., 2019a; Fan et al., 2020) (shown in Figure 3A).

**TABLE 2:** White-matter functional networks.

Name	White-matter network	Layer	Correlation with gray-matter network (*r* value)
WM1	Superior longitudinal fasciculus network	Superficial	Dorsal attention network (0.73)
WM2	Inferior longitudinal fasciculus network	Superficial	–
WM3	Anterior corpus callosum network	Superficial	Salience/Ventral attention (0.46)
WM4	Corpus callosum network	Middle	Salience/Ventral attention (0.77)
WM5	Occipital network	Superficial	Visual network (0.87)
WM6	Inferior temporal network	Superficial	Sensor-motor network (0.73)
WM7	Prefrontal network	Superficial	Default-mode network (0.78)
WM8	Superior corona radiate network	Middle	–
WM9	Post-central network	Superficial	Sensor-motor network (0.87)
WM10	Cerebellar network	Superficial	–
WM11	Posterior callosum network	Middle	Default-mode network (0.78)
WM12	Inferior frontoparietal network	Superficial	Control network (0.91)
WM13	Deep network	Deep	–

‘–’ implies that the value of functional connectivity below 0.4 (r < 0.4).

### Group differences of functional connectivity between white-matter functional networks

**FIGURE 4:**
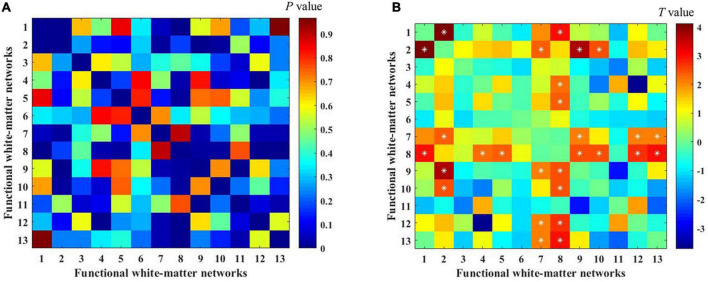
Differences in functional connectivity between myotonic dystrophy type 1 (DM1) and healthy controls (HCs). **(A,B)** The color bar showed the *P*-value and the *T*-value separately [two-sample *t*-tests with 1000 permutations, *p* < 0.05, network-based statistic (NBS) corrected]. *represents significant differences after NBS corrected.

To investigate the differences in white-matter networks between DM1 and HCs, we adopted a two-sample *t*-test for the functional connectivity within white-matter functional networks between the two groups. We identified that the between-network connectivity of DM1 was significantly higher than HCs (*p* < 0.05, NBS corrected; Figure 4). To better illustrate FC patterns of abnormalities in DM1, we summarize the increased FC in both intra-layer networks and inter-layer networks (shown in Figure 3B). The connections of intra-layer networks are mainly involved in adjacent WMs. They thus were named as short-range connectivity. Long-range connectivity comprised connections that linked superficial networks with middle networks and with deep networks or between the latter two. Anatomically speaking, the superficial white-matter networks may interact indirectly through the gray-matter network, while the middle and deep white-matter networks communicate more directly through axon-axon interactions.

As for intra-layer networks, within superficial networks, results showed that DM1 had increased functional connectivity mainly related to the inferior longitudinal fasciculus network (WM2) and prefrontal network (WM7). The abnormalities of paired interactions included WM2–WM1 (superior longitudinal fasciculus network), WM2–WM9 (post-central network), W2–WM10 (cerebellar network), and WM2–WM7, WM7–WM9, and WM7-WM12 (inferior frontoparietal network). Meanwhile, within middle networks, DM1 also showed increased FC between the corpus callosum network (WM4) and WM8 (superior corona radiata network). In addition, we observed increased FC in inter-layer networks. Specifically, between superficial and middle networks, we found increased FC mainly located in the superior corona radiate network (WM8), which showed abnormal interactions with the superior longitudinal fasciculus network (WM1), occipital network (WM5), post-central network (WM9), cerebellar network (WM10), and inferior frontoparietal network (WM12). We also demonstrated that DM1 had increased functional connectivity not only between deep and superficial networks but also in the middle network, and the former was mainly located in the prefrontal network (WM7), the latter was mainly located in the superior corona radiata network (WM8). No significant decrease in functional connections was observed. DM1 showed widespread dysfunctions across three layers within white-matter functional networks (shown in Figure 3B).

### Correlations of abnormal functional connectivity with clinical variables

After false discovery rate (FDR) correction for multiple comparisons, we failed to find significant correlations between abnormal functional connectivity and the MMSE or HAMD scores.

## Discussion

In this study, we identified 13 white-matter functional networks consisting of three layers (superficial, middle, and deep) by using a clustering method, which is consistent with previous studies (Peer et al., 2017; Jiang et al., 2019a,b, 2020; Lu et al., 2021; Li et al., 2022). The interactions between these white-matter functional networks were estimated by FC. Compared to HCs, DM1 showed increased FC in both intra-layer and inter-layer white-matter networks. Specifically, for intra-layer white-matter networks, DM1 had increased FC within superficial white-matter networks mainly related to the default mode network and the perception-motor network. We also found increased FC in inter-layer white-matter networks (dominated by middle and deep networks). The widely distributed abnormalities of white-matter functional networks provide a new perspective to understand the underlying intricated pathological mechanism of DM1. This echoed previous evidence that DM1 patients showed widespread white-matter alterations (Minnerop et al., 2011; Wozniak et al., 2011; Serra et al., 2015).

The disturbing connection of intra-layer networks was mainly related to three white-matter networks, namely, the inferior longitudinal fasciculus network (WM2), prefrontal network (WM7) related to the default-mode network, and post-central network (WM9) related to the perception-motor network. A most significantly increased FC was observed between the inferior longitudinal fasciculus network (WM2) and the superior longitudinal fasciculus network (WM1). The inferior longitudinal fasciculus (ILF) plays an important role in a wide range of brain functions related to the visual modality, including object recognition, face recognition, reading, lexical and semantic processing, emotion processing, and visual memory (Herbet et al., 2018). The superior longitudinal fascicle (SLF) is a major intrahemispheric fiber tract that connects the occipital, parietal, and temporal lobes with the frontal cortex (Schmahmann et al., 2008; Kamali et al., 2014; Ramos-Fresnedo et al., 2019). It is a multi-functional white-matter pathway mainly involved in the function of language, attention, memory, and emotions (Kamali et al., 2014). The present finding that increased FC between WM1 and WM2 might suggest the corresponding cognitive function of DM1 patients is impaired. Previous DTI studies have also reported the reduced fractional anisotropy (FA) of the inferior and superior longitudinal fascicle in DM1 patients, and the alterations of FA were correlated with genetic, clinical disability, and global cognitive performance (Serra et al., 2015; Okkersen et al., 2017). The prefrontal cortex is strongly implicated in higher-level cognitive and behavior functions (Ruby and Decety, 2003; Ranganath and Jacob, 2016). DM1 patients showed more connections between prefrontal and cerebellar regions that were also highlighted by one study before, which identified the relationship between these regions and the severity of patients’ deficits in cognitive function (i.e., Theory of Mind) (Serra et al., 2016). Similarly, in DM1, increased FC was also found in critical areas of the default mode network correlated with the presence of atypical personality traits that may account for overall cognitive dysfunction (Serra et al., 2014). During motor task-evoked fMRI studies, DM1 patients also showed greater activation in sensorimotor areas (Caramia et al., 2010; Toth et al., 2015). Moreover, the increased FC of superficial perception-motor networks has been found in other brain disorders, especially schizophrenia (Jiang et al., 2019a). In addition, increased FC also occurred in middle layer networks between the corpus callosum network (WM4) and superior corona radiata network (WM8). DTI studies confirmed reduced volume of the corpus callosum in myotonic dystrophy patients (Ota et al., 2006; Cabada et al., 2017). One study reported that corona radiate might be associated with executive function, attention, and processing of emotions (Yin et al., 2013). Zanigni et al. (2016) also found correlations between alterations of the posterior part of the corpus callosum and posterior corona radiate and the global motor and cognitive function (measured by MMSE score) in DM1. Therefore, we speculated that the enhanced FC within intra-layer networks might be associated with primary motor impairment and the higher-level cognitive dysfunction in DM1, and further study is needed to explore the relationship.

Besides, increased connections of DM1 patients also occurred within inter-layer WM networks, for instance, increased FC between deep and superficial networks (the prefrontal network, WM7) and middle networks (superior corona radiata network, WM8). Notably, all increased FC between middle and superficial networks were associated with a superior corona radiata network. Combined with the changes of FC within the inter-layer networks, it further authenticates the importance of the prefrontal network and the corona radiata network in DM1. In the DM1 animal model, the prefrontal cortex was mainly observed for the anatomical distribution of dopaminergic dysfunction (Ramon-Duaso et al., 2019). Other studies demonstrated that the prefrontal cortex might be related to maintaining and manipulating cognitive information (Quinette et al., 2006; Marchetti, 2014). The alterations across inter-layer suggested dysfunctional interactions between white-matter functional networks in DM1. The enhanced functional connectivity might suggest an insufficient or ineffective informational integration across white-matter functional networks. Meanwhile, these data support a possible fundamental mechanism that the increased brain connectivity might explain the high-level cognitive manifestations observed in DM1 patients.

A previous study revealed a less distinct WM/GM boundary in patients with autism, and it would bring the confusion of WM and GM cells in the boundary (Andrews et al., 2017). It also proposed that the fMRI signals of adjacent white-matter/gray-matter voxels could be confused, and the functional covariance between white-matter and gray-matter regions near the boundary (Chen et al., 2021). In the present study, in addition to the identified alterations of white-matter functional connectivity in DM1 patients, we also examined the interactions between white-matter and gray-matter networks. Similar to previous results, the correlation between deep white-matter network and gray-matter were weaker than the superficial white-matter networks (Peer et al., 2017; Jiang et al., 2019a). A study that measured the hemodynamic response function (HRF) of white-matter also found that in superficial white-matter voxels, the HRF showed a shape more similar to that of gray-matter (Li et al., 2019b). The explanation of the phenomena might be that superficial white-matter tracts connect distant cortical neuron cell bodies engaged in different functions, whereas deep white-matter tracts are less surrounded by gray-matter (Peer et al., 2017; Ding et al., 2018; Fan et al., 2020).

To validate and extend our findings on the functional connectivity of white-matter networks in DM1 patients, we also performed a voxel-based morphological (VBM) analysis to evaluate the alteration of white-matter in structure. The results of these voxel-based analyzes are provided in Supplementary material. Our results were in line with previous reports showing that white-matter atrophy was observed in DM1 patients in the splenium of the corpus callosum and middle cerebellar peduncle (Minnerop et al., 2011; Meola and Cardani, 2015; Schneider-Gold et al., 2015). Moreover, one DTI study found a significant correlation between visuo-spatial deficit and posterior corpus callosum (Cabada et al., 2017), and another DTI study found that the cognitive performance in visuomotor coordination and working memory tasks was associated with microstructural damage of corpus callosum (Baldanzi et al., 2016). Together, these studies demonstrate that corpus callosum atrophy might be associated with worse cognitive function in DM1 patients. Besides, we also found that compared to HCs, the volume of the posterior limb of the internal capsule increased in DM1 patients. A study focused on assessing alteration in spontaneous neural activity of the sensorimotor network in patients with DM1 found that patients with DM1 had increased power spectral density (PSD) in the anterior and posterior limbs of the internal capsule, which is associated with the motor function (Park et al., 2018). Anatomically, the anterior half of the posterior limb of the internal capsule contains the corticospinal tract, corticorubral tract, and corticopontine tract (Emos and Agarwal, 2021). And these fibers play a major role in the coordination of voluntary motor functions (Tredici et al., 1990). Therefore, the abnormality of the posterior limb of the internal capsule in DM1 patients might be related to the impaired motor performance of DM1 patients.

The present study utilized the k-means clustering method, which has been widely used in the fMRI data. However, considering the high dimension of the fMRI data, studies have proposed advanced methods, such as the regression mixture modeling approach to cluster fMRI time series (Oikonomou and Blekas, 2013; Oikonomou et al., 2020). The new method achieved very promising results in both simulated and real fMRI data. And the excellent performance in resting-state networks (RSNs) reminds us that it could be a useful tool to reveal the mechanism of DM1 I in future studies.

Although we did not find abnormal network connections associated with clinical variables after correction, our studies showed extensively increased FC across intra-layer and inter-layer white-matter functional networks. One possible explanation is that the cognitive and behavioral manifestations of the DM1 are the result of multiple white-matter network layer-integration changes rather than attributed to abnormalities of the interactions between any two white-matter networks alone. The MMSE and HAMD-17 scores focus on broader cognitive and emotional domains. Future studies should target more specific cognitive processes.

Overall, the current findings further demonstrated the relevance between superficial white-matter networks and gray-matter networks and provided evidence for the dysfunctional connectivity of Myotonic Dystrophy Type 1 in the white-matter function network perspective. Moreover, these findings may contribute to understand the pathophysiology of DM1 and may guide the therapeutics development in the future.

## Limitations

Our study had several limitations. First, we obtained a relatively small sample size due to the relative scarcity of the disease, which may also explain our failure to find abnormal functional connectivity correlations with clinical variables in our study. Future investigators must utilize a larger sample to verify the stability and reproducibility of the results. Second, our results were limited to cross-sectional comparisons, and the participants were adults. Considering the finding showed that DM1 may be a neurodegenerative disease, future researchers could pay more attention to the variation of abnormalities in the white-matter functional network in participants with DM1 at different ages. Third, a lesion mask would be applied in the preprocessing to avoid bias in the tissue segmentation in the future study.

## Conclusion

In the current study, we investigated interactions between white-matter functional networks in DM1. The current findings uncovered increased inter-layer and intra-layer interactions across superficial, middle, and deep white-matter networks, tentatively providing additional information that the pathophysiology of DM1 may be related to white-matter functional networks engaged in sensory-motor perception and cognitive functions. To some extent, the widespread disrupted white-matter networks can underlie cognitive-behavioral dysfunctions in DM1. In general, widespread dysfunction of white-matter in DM1 may be accountable for its pathological mechanism. Our findings supported and extended impairments of white-matter dysfunction in DM1.

## Data availability statement

The raw data supporting the conclusions of this article will be made available by the authors, without undue reservation.

## Ethics statement

The studies involving human participants were reviewed and approved by Ethic Committee of Ruijin Hospital Affiliated to Shanghai Jiao Tong University School of Medicine. The patients/participants provided their written informed consent to participate in this study.

## Author contributions

X-ZJ, H-YZ, D-QL, JeL, and JnL designed and conceptualized the study. PH participated in data collection. JnL and JeL analyzed the data and wrote the manuscript. X-ZJ, H-YZ, and D-QL supervised procedures of the study. JnL, JeL, L-NH, Q-GD, LZ, ML, JZ, HZ, LC, HL, X-ZJ, H-YZ, and D-QL revised the manuscript. All authors contributed to the final version of the manuscript.

## Footnotes

1
http://rfmri.org/dpabi

2
http://mind.huji.ac.il/white-matter.aspx

## References

[B1] Andrews D. S. Avino T. A. Gudbrandsen M. Daly E. Marquand A. Murphy C. M. (2017). *In Vivo* Evidence of Reduced Integrity of the Gray-White Matter Boundary in Autism Spectrum Disorder. *Cereb. Cortex* 27 877–887. 10.1093/cercor/bhw404 28057721 PMC6093436

[B2] Ashburner J. (2007). A fast diffeomorphic image registration algorithm. *NeuroImage* 38 95–113. 10.1016/j.neuroimage.2007.07.007 17761438

[B3] Baldanzi S. Cecchi P. Fabbri S. Pesaresi I. Simoncini C. Angelini C. (2016). Relationship between neuropsychological impairment and grey and white matter changes in adult-onset myotonic dystrophy type 1. *NeuroImage Clin.* 12 190–197. 10.1016/j.nicl.2016.06.011 27437180 PMC4939389

[B4] Behzadi Y. Restom K. Liau J. Liu T. T. (2007). A component based noise correction method (CompCor) for BOLD and perfusion based fMRI. *Neuroimage* 37 90–101. 10.1016/j.neuroimage.2007.04.042 17560126 PMC2214855

[B5] Biswal B. Zerrin Yetkin F. Haughton V. M. Hyde J. S. (1995). Functional connectivity in the motor cortex of resting human brain using echo-planar mri. *Magn. Reson. Med.* 34 537–541. 10.1002/mrm.1910340409 8524021

[B6] Bu X. Liang K. Lin Q. Gao Y. Qian A. Chen H. (2020). Exploring white matter functional networks in children with attention-deficit/hyperactivity disorder. *Brain Commun.* 2:fcaa113. 10.1093/braincomms/fcaa113 33215081 PMC7660033

[B7] Buckner R. L. Krienen F. M. Castellanos A. Diaz J. C. Yeo B. T. T. (2011). The organization of the human cerebellum estimated by intrinsic functional connectivity. *J. Neurophysiol.* 106 2322–2345. 10.1152/jn.00339.2011 21795627 PMC3214121

[B8] Cabada T. Iridoy M. Jericó I. Lecumberri P. Seijas R. Gargallo A. (2017). Brain Involvement in Myotonic Dystrophy Type 1: a Morphometric and Diffusion Tensor Imaging Study with Neuropsychological Correlation. *Arch. Clin. Neuropsychol.* 32 401–412. 10.1093/arclin/acx008 28164212

[B9] Caballero-Gaudes C. Reynolds R. C. (2017). Methods for cleaning the BOLD fMRI signal. *Neuroimage* 154 128–149. 10.1016/j.neuroimage.2016.12.018 27956209 PMC5466511

[B10] Caramia F. Mainero C. Gragnani F. Tinelli E. Fiorelli M. Ceschin V. (2010). Functional MRI changes in the central motor system in myotonic dystrophy type 1. *Magn. Reson. Imaging* 28 226–234. 10.1016/j.mri.2009.07.006 19695817

[B11] Caso F. Agosta F. Peric S. Rakočević-Stojanović V. Copetti M. Kostic V. S. (2014). Cognitive Impairment in Myotonic Dystrophy Type 1 Is Associated with White Matter Damage. *PLoS One* 9:e104697. 10.1371/journal.pone.0104697 25115999 PMC4130603

[B12] Chen H. Long J. Yang S. He B. (2021). Atypical Functional Covariance Connectivity Between Gray and White Matter in Children With Autism Spectrum Disorder. *Autism Res.* 14 464–472. 10.1002/aur.2435 33206448

[B13] Craddock R. C. James G. A. Holtzheimer P. E. Hu X. P. Mayberg H. S. (2012). A whole brain fMRI atlas generated *via* spatially constrained spectral clustering. *Hum. Brain Mapp.* 33 1914–1928. 10.1002/hbm.21333 21769991 PMC3838923

[B14] Desikan R. S. Ségonne F. Fischl B. Quinn B. T. Dickerson B. C. Blacker D. (2006). An automated labeling system for subdividing the human cerebral cortex on MRI scans into gyral based regions of interest. *NeuroImage* 31 968–980. 10.1016/j.neuroimage.2006.01.021 16530430

[B15] Ding Z. Huang Y. Bailey S. K. Gao Y. Cutting L. E. Rogers B. P. (2018). Detection of synchronous brain activity in white matter tracts at rest and under functional loading. *Proc. Natl. Acad. Sci. U.S.A.* 115 595–600. 10.1073/pnas.1711567115 29282320 PMC5776967

[B16] Ding Z. Newton A. T. Xu R. Anderson A. W. Morgan V. L. Gore J. C. (2013). Spatio-temporal correlation tensors reveal functional structure in human brain. *PLoS One* 8:e82107. 10.1371/journal.pone.0082107 24339997 PMC3855380

[B17] Ding Z. Xu R. Bailey S. K. Wu T.-L. Morgan V. L. Cutting L. E. (2016). Visualizing functional pathways in the human brain using correlation tensors and magnetic resonance imaging. *Magn. Reson. Imaging* 34 8–17. 10.1016/j.mri.2015.10.003 26477562 PMC4714593

[B18] Emery A. E. H. (1991). Population frequencies of inherited neuromuscular diseases—A world survey. *Neuromuscul. Disord.* 1 19–29. 10.1016/0960-8966(91)90039-U 1822774

[B19] Emos M. C. Agarwal S. (2021). *Neuroanatomy, Internal Capsule.* StatPearls Publishing Available online at: https://www.ncbi.nlm.nih.gov/books/NBK542181/ (accessed July 5, 2022). 31194338

[B20] Fabri M. Polonara G. (2013). Functional topography of human corpus callosum: an FMRI mapping study. *Neural Plast.* 2013:251308. 10.1155/2013/251308 23476810 PMC3586479

[B21] Fabri M. Polonara G. Mascioli G. Salvolini U. Manzoni T. (2011). Topographical organization of human corpus callosum: an fMRI mapping study. *Brain Res.* 1370 99–111. 10.1016/j.brainres.2010.11.039 21081115

[B22] Fan Y. Li Z. Duan X. Xiao J. Guo X. Han S. (2020). Impaired interactions among white-matter functional networks in antipsychotic-naive first-episode schizophrenia. *Hum. Brain Mapp.* 41 230–240. 10.1002/hbm.24801 31571346 PMC7267955

[B23] Friston K. J. Williams S. Howard R. Frackowiak R. S. J. Turner R. (1996). Movement-Related effects in fMRI time-series: movement Artifacts in fMRI. *Magn. Reson. Med.* 35 346–355. 10.1002/mrm.1910350312 8699946

[B24] Gallais B. Montreuil M. Gargiulo M. Eymard B. Gagnon C. Laberge L. (2015). Prevalence and correlates of apathy in myotonic dystrophy type 1. *BMC Neurol.* 15:148. 10.1186/s12883-015-0401-6 26296336 PMC4546188

[B25] Gawryluk J. R. Mazerolle E. L. Brewer K. D. Beyea S. D. D’Arcy R. C. N. (2011). Investigation of fMRI activation in the internal capsule. *BMC Neurosci.* 12:56. 10.1186/1471-2202-12-56 21672250 PMC3141570

[B26] Gawryluk J. R. Mazerolle E. L. D’Arcy R. C. N. (2014). Does functional MRI detect activation in white matter? A review of emerging evidence, issues, and future directions. *Front. Neurosci.* 8:239. 10.3389/fnins.2014.00239 25152709 PMC4125856

[B27] Gourdon G. Meola G. (2017). Myotonic Dystrophies: state of the Art of New Therapeutic Developments for the CNS. *Front. Cell. Neurosci.* 11:101. 10.3389/fncel.2017.00101 28473756 PMC5397409

[B28] Herbet G. Zemmoura I. Duffau H. (2018). Functional Anatomy of the Inferior Longitudinal Fasciculus: from Historical Reports to Current Hypotheses. *Front. Neuroanat.* 12:77. 10.3389/fnana.2018.00077 30283306 PMC6156142

[B29] Huang Y. Bailey S. K. Wang P. Cutting L. E. Gore J. C. Ding Z. (2018). Voxel-wise detection of functional networks in white matter. *NeuroImage* 183 544–552. 10.1016/j.neuroimage.2018.08.049 30144573 PMC6226032

[B30] Ji G.-J. Liao W. Chen F.-F. Zhang L. Wang K. (2017). Low-frequency blood oxygen level-dependent fluctuations in the brain white matter: more than just noise. *Sci. Bull.* 62 656–657. 10.1016/j.scib.2017.03.021 36659309

[B31] Jiang Y. Song L. Li X. Zhang Y. Chen Y. Jiang S. (2019b). Dysfunctional white-matter networks in medicated and unmedicated benign epilepsy with centrotemporal spikes. *Hum. Brain Mapp.* 40 3113–3124. 10.1002/hbm.24584 30937973 PMC6865396

[B32] Jiang Y. Luo C. Li X. Li Y. Yang H. Li J. (2019a). White-matter functional networks changes in patients with schizophrenia. *NeuroImage* 190 172–181. 10.1016/j.neuroimage.2018.04.018 29660513

[B33] Jiang Y. Yao D. Zhou J. Tan Y. Huang H. Wang M. (2020). Characteristics of disrupted topological organization in white matter functional connectome in schizophrenia. *Psychol. Med.* 52 1333–1343. 10.1017/S0033291720003141 32880241

[B34] Kamali A. Flanders A. E. Brody J. Hunter J. V. Hasan K. M. (2014). Tracing superior longitudinal fasciculus connectivity in the human brain using high resolution diffusion tensor tractography. *Brain Struct. Funct.* 219 269–281. 10.1007/s00429-012-0498-y 23288254 PMC3633629

[B35] Li M. Newton A. T. Anderson A. W. Ding Z. Gore J. C. (2019b). Characterization of the hemodynamic response function in white matter tracts for event-related fMRI. *Nat. Commun.* 10:1140. 10.1038/s41467-019-09076-2 30850610 PMC6408456

[B36] Li J. Biswal B. B. Wang P. Duan X. Cui Q. Chen H. (2019a). Exploring the functional connectome in white matter. *Hum. Brain Mapp.* 40 4331–4344. 10.1002/hbm.24705 31276262 PMC6865787

[B37] Li J. Chen H. Fan F. Qiu J. Du L. Xiao J. (2020). White-matter functional topology: a neuromarker for classification and prediction in unmedicated depression. *Transl. Psychiatry* 10:365. 10.1038/s41398-020-01053-4 33127899 PMC7603321

[B38] Li X. Jiang Y. Li W. Qin Y. Li Z. Chen Y. (2022). Disrupted functional connectivity in white matter resting-state networks in unilateral temporal lobe epilepsy. *Brain Imaging Behav.* 16 324–335. 10.1007/s11682-021-00506-8 34478055

[B39] Logothetis N. K. Pauls J. Augath M. Trinath T. Oeltermann A. (2001). Neurophysiological investigation of the basis of the fMRI signal. *Nature* 412 150–157. 10.1038/35084005 11449264

[B40] Lopez-Titla M. M. Chirino A. Cruz Solis S. V. Hernandez-Castillo C. R. Diaz R. Márquez-Quiroz L. D. C. (2021). Cognitive Decline and White Matter Integrity Degradation in Myotonic Dystrophy Type I. *J. Neuroimaging* 31 192–198. 10.1111/jon.12786 32936994

[B41] Lu F. Cui Q. He Z. Tang Q. Chen Y. Sheng W. (2021). Superficial white-matter functional networks changes in bipolar disorder patients during depressive episodes. *J. Affect. Disord.* 289 151–159. 10.1016/j.jad.2021.04.029 33984685

[B42] Marchetti G. (2014). Attention and working memory: two basic mechanisms for constructing temporal experiences. *Front. Psychol.* 5:880. 10.3389/fpsyg.2014.00880 25177305 PMC4132481

[B43] Marussich L. Lu K.-H. Wen H. Liu Z. (2017). Mapping white-matter functional organization at rest and during naturalistic visual perception. *Neuroimage* 146 1128–1141. 10.1016/j.neuroimage.2016.10.005 27720819 PMC5321894

[B44] Meda S. A. Ruano G. Windemuth A. O’Neil K. Berwise C. Dunn S. M. (2014). Multivariate analysis reveals genetic associations of the resting default mode network in psychotic bipolar disorder and schizophrenia. *Proc. Natl. Acad. Sci. U.S.A.* 111 E2066–E2075. 10.1073/pnas.1313093111 24778245 PMC4024891

[B45] Meola G. Cardani R. (2015). Myotonic dystrophies: an update on clinical aspects, genetic, pathology, and molecular pathomechanisms. *Biochim. Biophys. Acta* 1852 594–606. 10.1016/j.bbadis.2014.05.019 24882752

[B46] Meola G. Sansone V. (2007). Cerebral involvement in myotonic dystrophies. *Muscle Nerve* 36 294–306. 10.1002/mus.20800 17486579

[B47] Meola G. Sansone V. Radice S. Skradski S. Ptacek L. (1996). A family with an unusual myotonic and myopathic phenotype and no CTG expansion (proximal myotonic myopathy syndrome): a challenge for future molecular studies. *Neuromuscul. Disord.* 6 143–150. 10.1016/0960-8966(95)00040-2 8784800

[B48] Minnerop M. Weber B. Schoene-Bake J.-C. Roeske S. Mirbach S. Anspach C. (2011). The brain in myotonic dystrophy 1 and 2: evidence for a predominant white matter disease. *Brain* 134 3530–3546. 10.1093/brain/awr299 22131273 PMC3235566

[B49] Modoni A. Silvestri G. Vita M. G. Quaranta D. Tonali P. A. Marra C. (2008). Cognitive impairment in myotonic dystrophy type 1 (DM1): a longitudinal follow-up study. *J. Neurol.* 255 1737–1742. 10.1007/s00415-008-0017-5 18821050

[B50] Oikonomou V. P. Blekas K. (2013). An Adaptive Regression Mixture Model for fMRI Cluster Analysis. *IEEE Trans. Med. Imaging* 32 649–659. 10.1109/TMI.2012.2221731 23047865

[B51] Oikonomou V. P. Blekas K. Astrakas L. (2020). Identification of Brain Functional Networks Using a Model-Based Approach. *Int. J. Patt. Recogn. Artif. Intell.* 34:2057004. 10.1142/S0218001420570049

[B52] Okkersen K. Monckton D. G. Le N. Tuladhar A. M. Raaphorst J. van Engelen B. G. M. (2017). Brain imaging in myotonic dystrophy type 1: a systematic review. *Neurology* 89 960–969. 10.1212/WNL.0000000000004300 28768849

[B53] Oldfield R. C. (1971). The assessment and analysis of handedness: the Edinburgh inventory. *Neuropsychologia* 9 97–113. 10.1016/0028-3932(71)90067-4 5146491

[B54] Ota M. Sato N. Ohya Y. Aoki Y. Mizukami K. Mori T. (2006). Relationship between diffusion tensor imaging and brain morphology in patients with myotonic dystrophy. *Neurosci. Lett.* 407 234–239. 10.1016/j.neulet.2006.08.077 16978781

[B55] Park J.-S. Seo J. Cha H. Song H.-J. Lee S.-H. Jang K. E. (2018). Altered power spectral density in the resting-state sensorimotor network in patients with myotonic dystrophy type 1. *Sci. Rep.* 8:987. 10.1038/s41598-018-19217-0 29343751 PMC5772436

[B56] Peer M. Nitzan M. Bick A. S. Levin N. Arzy S. (2017). Evidence for Functional Networks within the Human Brain’s White Matter. *J. Neurosci.* 37 6394–6407. 10.1523/JNEUROSCI.3872-16.2017 28546311 PMC6596606

[B57] Power J. D. Barnes K. A. Snyder A. Z. Schlaggar B. L. Petersen S. E. (2012). Spurious but systematic correlations in functional connectivity MRI networks arise from subject motion. *NeuroImage* 59 2142–2154. 10.1016/j.neuroimage.2011.10.018 22019881 PMC3254728

[B58] Quinette P. Guillery-Girard B. Noël A. de la Sayette V. Viader F. Desgranges B. (2006). The relationship between working memory and episodic memory disorders in transient global amnesia. *Neuropsychologia* 44 2508–2519. 10.1016/j.neuropsychologia.2006.03.031 16697428

[B59] Rakocevic-Stojanovic V. Peric S. Madzarevic R. Dobricic V. Ralic V. Ilic V. (2014). Significant impact of behavioral and cognitive impairment on quality of life in patients with myotonic dystrophy type 1. *Clin. Neurol. Neurosurg.* 126 76–81. 10.1016/j.clineuro.2014.08.021 25215445

[B60] Ramon-Duaso C. Gener T. Consegal M. Fernández-Avilés C. Gallego J. J. Castarlenas L. (2019). Methylphenidate Attenuates the Cognitive and Mood Alterations Observed in Mbnl2 Knockout Mice and Reduces Microglia Overexpression. *Cereb. Cortex* 29 2978–2997. 10.1093/cercor/bhy164 30060068 PMC7963113

[B61] Ramos-Fresnedo A. Segura-Duran I. Chaichana K. L. Pillai J. J. (2019). “Chapter 2 - Supratentorial White Matter Tracts,” in *Comprehensive Overview of Modern Surgical Approaches to Intrinsic Brain Tumors*, eds Chaichana K. Quiñones-Hinojosa A. (Cambridge: Academic Press), 23–35. 10.1016/B978-0-12-811783-5.00002-1

[B62] Ranganath A. Jacob S. N. (2016). Doping the Mind: dopaminergic Modulation of Prefrontal Cortical Cognition. *Neuroscientist* 22 593–603. 10.1177/1073858415602850 26338491

[B63] Ruby P. Decety J. (2003). What you believe versus what you think they believe: a neuroimaging study of conceptual perspective-taking: a PET study of conceptual perspective-taking. *Eur. J. Neurosci.* 17 2475–2480. 10.1046/j.1460-9568.2003.02673.x 12814380

[B64] Schmahmann J. D. Smith E. E. Eichler F. S. Filley C. M. (2008). Cerebral white matter: neuroanatomy, clinical neurology, and neurobehavioral correlates. *Ann. N.Y. Acad. Sci.* 1142 266–309. 10.1196/annals.1444.017 18990132 PMC3753195

[B65] Schneider-Gold C. Bellenberg B. Prehn C. Krogias C. Schneider R. Klein J. (2015). Cortical and Subcortical Grey and White Matter Atrophy in Myotonic Dystrophies Type 1 and 2 Is Associated with Cognitive Impairment Depression and Daytime Sleepiness. *PLoS One* 10:e0130352. 10.1371/journal.pone.0130352 26114298 PMC4482602

[B66] Serra L. Mancini M. Silvestri G. Petrucci A. Masciullo M. Spanò B. (2016). Brain Connectomics’ Modification to Clarify Motor and Nonmotor Features of Myotonic Dystrophy Type 1. *Neural Plast.* 2016:2696085. 10.1155/2016/2696085 27313901 PMC4897716

[B67] Serra L. Petrucci A. Spanò B. Torso M. Olivito G. Lispi L. (2015). How genetics affects the brain to produce higher-level dysfunctions in myotonic dystrophy type 1. *Funct. Neurol.* 30 21–31. 26214024 PMC4520669

[B68] Serra L. Silvestri G. Petrucci A. Basile B. Masciullo M. Makovac E. (2014). Abnormal Functional Brain Connectivity and Personality Traits in Myotonic Dystrophy Type 1. *JAMA Neurol.* 71:603. 10.1001/jamaneurol.2014.130 24664202

[B69] Toth A. Lovadi E. Komoly S. Schwarcz A. Orsi G. Perlaki G. (2015). Cortical involvement during myotonia in myotonic dystrophy: an fMRI study. *Acta Neurol. Scand.* 132 65–72. 10.1111/ane.12360 25630356

[B70] Tredici G. Barajon I. Pizzini G. Sanguineti I. (1990). The organization of corticopontine fibres in man. *Acta Anat.* 137 320–323. 10.1159/000146902 2368586

[B71] Van Dijk K. R. A. Sabuncu M. R. Buckner R. L. (2012). The influence of head motion on intrinsic functional connectivity MRI. *NeuroImage* 59 431–438. 10.1016/j.neuroimage.2011.07.044 21810475 PMC3683830

[B72] Wozniak J. R. Mueller B. A. Lim K. O. Hemmy L. S. Day J. W. (2014). Tractography reveals diffuse white matter abnormalities in Myotonic Dystrophy Type 1. *J. Neurol. Sci.* 341 73–78. 10.1016/j.jns.2014.04.005 24768314 PMC4042407

[B73] Wozniak J. R. Mueller B. A. Ward E. E. Lim K. O. Day J. W. (2011). White matter abnormalities and neurocognitive correlates in children and adolescents with myotonic dystrophy type 1: a diffusion tensor imaging study. *Neuromuscul. Disord.* 21 89–96. 10.1016/j.nmd.2010.11.013 21169018 PMC3026055

[B74] Wu T.-L. Wang F. Li M. Schilling K. G. Gao Y. Anderson A. W. (2019). Resting-state white matter-cortical connectivity in non-human primate brain. *Neuroimage* 184 45–55. 10.1016/j.neuroimage.2018.09.021 30205207 PMC6250128

[B75] Wu X. Yang Z. Bailey S. K. Zhou J. Cutting L. E. Gore J. C. (2017). Functional connectivity and activity of white matter in somatosensory pathways under tactile stimulations. *NeuroImage* 152 371–380. 10.1016/j.neuroimage.2017.02.074 28284801 PMC5432381

[B76] Yin X. Han Y. Ge H. Xu W. Huang R. Zhang D. (2013). Inferior frontal white matter asymmetry correlates with executive control of attention. *Hum. Brain Mapp.* 34 796–813. 10.1002/hbm.21477 22110013 PMC6869851

[B77] Zalesky A. Fornito A. Bullmore E. T. (2010). Network-based statistic: identifying differences in brain networks. *NeuroImage* 53 1197–1207. 10.1016/j.neuroimage.2010.06.041 20600983

[B78] Zanigni S. Evangelisti S. Giannoccaro M. P. Oppi F. Poda R. Giorgio A. (2016). Relationship of white and gray matter abnormalities to clinical and genetic features in myotonic dystrophy type 1. *Neuroimage Clin.* 11 678–685. 10.1016/j.nicl.2016.04.012 27330968 PMC4900512

[B79] Zhang F. Li F. Yang H. Jin Y. Lai W. Roberts N. (2021a). Effect of experimental orthodontic pain on gray and white matter functional connectivity. *CNS Neurosci. Ther.* 27 439–448. 10.1111/cns.13557 33369178 PMC7941220

[B80] Zhang F. Yang Z. Qin K. Sweeney J. A. Roberts N. Jia Z. (2021b). Effect of jet lag on brain white matter functional connectivity. *Psychoradiology* 1 55–65. 10.1093/psyrad/kkaa003 38665361 PMC10917196

