# Supplementary material for: Increased functional connectivity of white-matter in myotonic dystrophy type 1
Source: Front Neurosci. 2022 Aug 1;16:953742. doi: 10.3389/fnins.2022.953742 (PMC9377538; doi:10.3389/fnins.2022.953742)
Supplement: Supplementary file 1 [file Data_Sheet_1.docx]

**Supplementary Table 1** Results of two-sample *t*-test from VBM analysis comparing DM1 and HC groups.

| **Regions (JHU-atlas)** | **Cluster size(voxels)** | **MNI Coordinates** | | | ***T* value** |
| --- | --- | --- | --- | --- | --- |
|  |  | **x** | **y** | **z** |  |
| Splenium_of_corpus_callosum | 42919 | -9 | -51 | 69 | -8.4925 |
| Middle_cerebellar_peduncle | 16089 | -6 | -7.5 | -4.5 | -7.1984 |
| Posterior_limb_of_internal_capsule_L | 459 | -9 | -9 | 6 | 7.011 |

*Note:* The statistical threshold was set at *p* < 0.05 for FDR (False Discovery Rate) corrected, minimum cluster size >100 voxels.


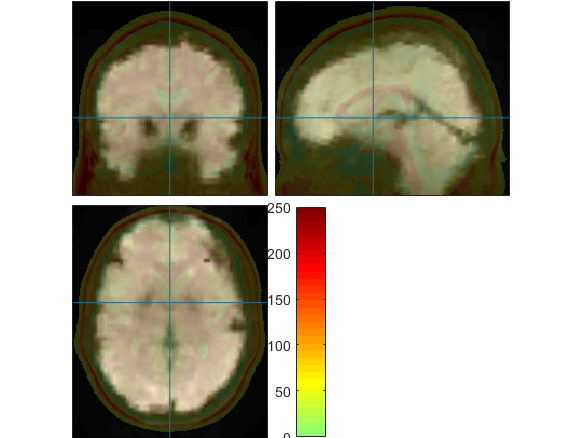


Figure S1. The segmentation effect map of sub02


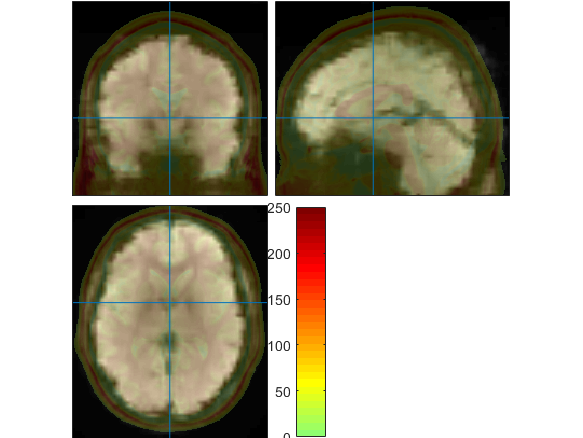


Figure S2. The segmentation effect map of sub03


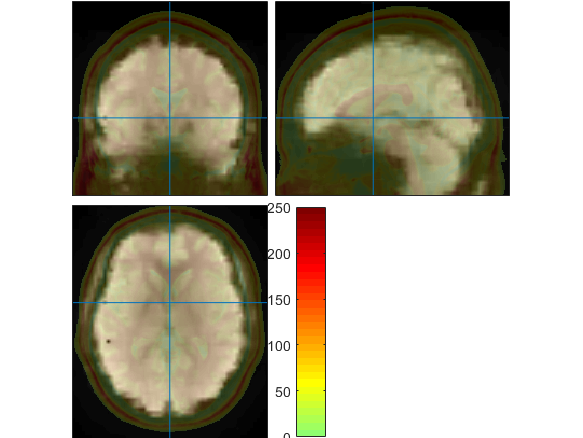


Figure S3. The segmentation effect map of sub04


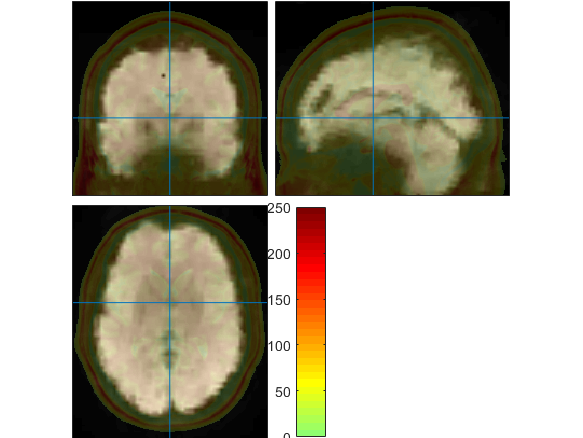


Figure S4. The segmentation effect map of sub07


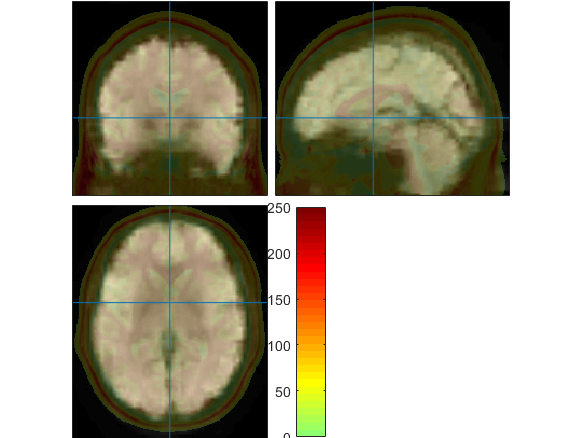


Figure S5. The segmentation effect map of sub10


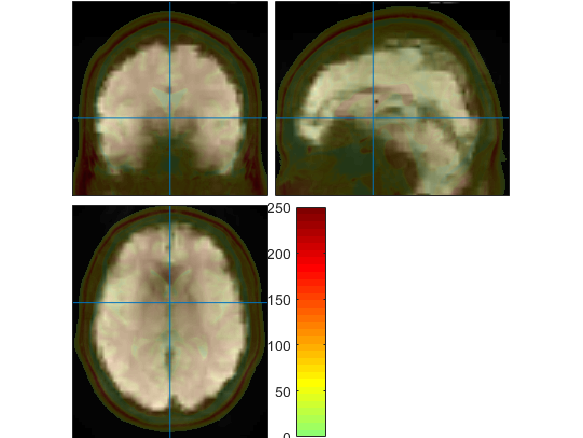


Figure S6. The segmentation effect map of sub15


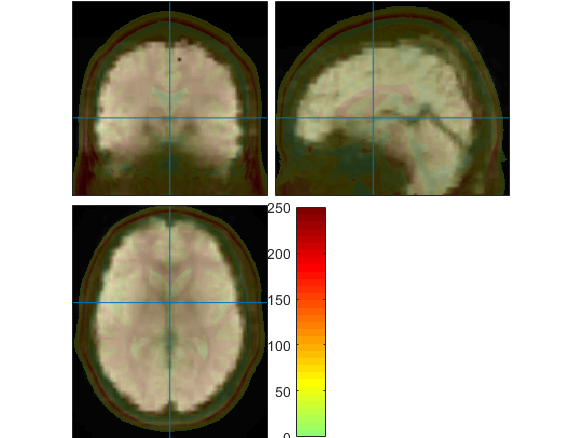


Figure S7. The segmentation effect map of sub17
